# Quantity and Quality of Inhaled Dose Predicts Immunopathology in Tuberculosis

**DOI:** 10.3389/fimmu.2015.00313

**Published:** 2015-06-29

**Authors:** Kevin P. Fennelly, Edward C. Jones-López

**Affiliations:** ^1^Department of Medicine, Emerging Pathogens Institute, University of Florida, Gainesville, FL, USA; ^2^Section of Infectious Diseases, Boston Medical Center, Boston University School of Medicine, Boston, MA, USA

**Keywords:** tuberculosis, inhaled dose, immunology, cough aerosol, latent infection, TB transmission

## Abstract

Experimental animal models of tuberculosis (TB) have convincingly demonstrated that inhaled dose predicts immunopathology and survival. In contrast, the importance of inhaled dose has generally not been appreciated in TB epidemiology, clinical science, or the practice of TB control. Infectiousness of TB patients has traditionally been assessed using microscopy for acid-fast bacilli in the sputum, which should be considered only a risk factor. We have recently demonstrated that cough aerosol cultures from index cases with pulmonary TB are the best predictors of new infection among household contacts. We suggest that cough aerosols of *M. tuberculosis* are the best surrogates of inhaled dose, and we hypothesize that the quantity of cough aerosols is associated with TB infection versus disease. Although several factors affect the quality of infectious aerosols, we propose that the particle size distribution of cough aerosols is an important predictor of primary upper airway disease and cervical lymphadenitis and of immune responses in exposed hosts. We hypothesize that large droplet aerosols (>5 μ) containing *M. tuberculosis* deposit in the upper airway and can induce immune responses without establishing infection. We suggest that this may partially explain the large proportion of humans who never develop TB disease in spite of having immunological evidence of *M. tuberculosis* infection (e.g., positive tuberculin skin test or interferon gamma release assay). If these hypotheses are proven true, they would alter the current paradigm of latent TB infection and reactivation, further demonstrating the need for better biomarkers or methods of assessing TB infection and the risk of developing disease.

## Introduction

In spite of the devastating impact of tuberculosis (TB) on mankind throughout history, scientists have struggled to decipher the determinants of the wide spectrum of infection and disease in TB. Data from over 50 years ago have suggested that the vast majority (90%) of persons infected with the causative agent, *Mycobacterium tuberculosis*, never manifest any symptoms or signs of disease. These persons are diagnosed with latent TB infection (LTBI) only by an immunological signature of infection using the traditional tuberculin skin test (TST) or a more modern interferon gamma release assay (IGRA) of the blood. It is estimated that one-third of the global population, or 2 billion people, are latently infected with TB. At the opposite end of the spectrum are persons who die of respiratory failure due to destruction of their lungs or who present with TB sepsis due to dissemination of *M. tuberculosis* in their bloodstream and to other organs. The variable balance between the immune response and virulence of the bacilli is responsible for the containment of *M. tuberculosis* in LTBI, for the destruction of lungs and other tissues due to an overly aggressive response, and for disseminated disease due to loss of control of *M. tuberculosis*. The actions of the immune system responsible for this variability are highly complex and are addressed by other authors in this issue. However, we present a simple concept that is poorly appreciated in both research and control of human TB: a major determinant of immunopathology in TB is the inhaled dose of *M. tuberculosis*. Our primary hypothesis is that small inhaled doses are more likely to result in LTBI, and that large inhaled doses are more likely to result in primary TB disease. In other words, we hypothesize that the quantity of inhaled dose predicts the immunopathology following infection. We propose that cough aerosol cultures of *M. tuberculosis* from human patients with active TB cases are better surrogates of inhaled dose than are sputa smears for acid-fast bacilli (AFB) or sputa cultures of *M. tuberculosis*.

We also propose that the quality of the infectious aerosol is an important determinant of immunopathology. The quality of an infectious aerosol includes its particle size distribution, virulence and infectivity of the pathogen, the duration of exposure to antimycobacterial drugs, desiccation, ultraviolet light, and other stressors. We will focus our discussion on the particle size distribution. We hypothesize that when *M. tuberculosis* is present in large droplet aerosols it deposits in the upper airways and produces immune responses but not necessarily infection. This would explain a large number of persons who are currently diagnosed as having LTBI but who never develop active TB disease. This idea also addresses the fundamental question of whether all those with positive TSTs or IGRAs are infected with viable *M. tuberculosis* (1). These hypotheses are based on our recent data on cough aerosols of *M. tuberculosis*; a new synthesis and interpretation of the literature on TB epidemiology, pathogenesis and clinical presentations; and principles of aerosol science. These concepts challenge current dogma in our understanding of TB, but we respectfully suggest that they help explain uncertainties in the science of TB and that they can be readily tested in the near future. If proven true, they would change the current paradigm of LTBI diagnosis and treatment. They would also emphasize the need for better biomarkers to confirm the presence of live bacilli within the human host and to predict the risk of reactivation of LTBI.

**Figure 1:**
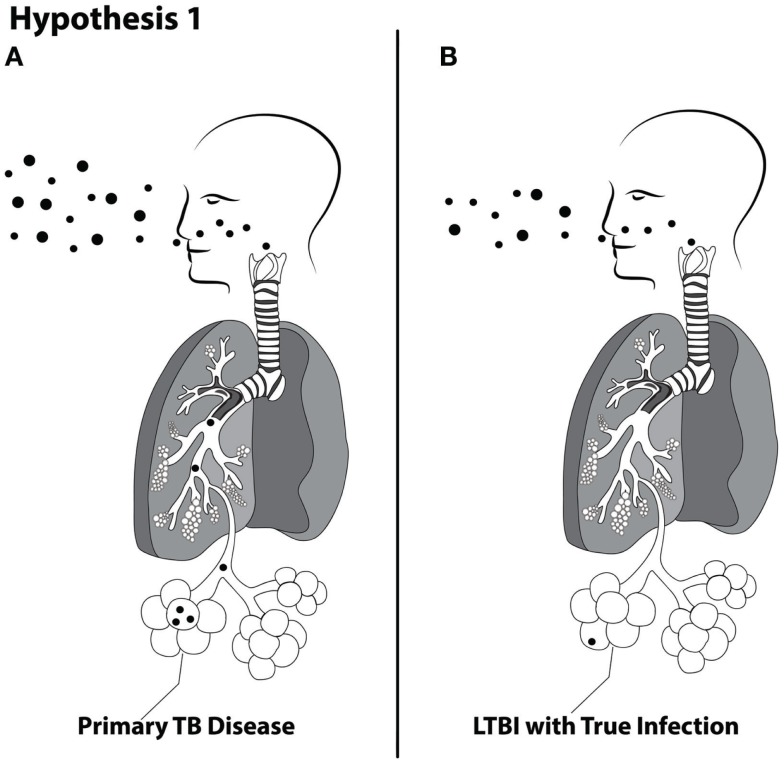
**Schematic of Hypothesis 1 demonstrating the proposed concept that larger inhaled doses are more likely to produce primary TB disease (A) and lower doses are more likely to produce latent TB infection (B)**.

Hypothesis 1. The magnitude of cough aerosol cultures in fine (≤5 μ) aerosols of *M. tuberculosis* from index cases of pulmonary TB is positively associated with TB disease (Figure 1).

(a) Large magnitudes of cough aerosol cultures are associated with primary TB.
(b) Small magnitudes of cough aerosol cultures are associated with reactivation TB within the first few years of exposure.

## Inhaled Dose–Response in Animal Models of TB

### General principle of the inoculum effect

A general principle in the study of infectious diseases is that the size of the inoculum, or dose, and virulence of a given pathogen predict the establishment of infection and subsequent pathogenesis in the host (2). This principle has been demonstrated in bacteria (3–5), fungi (6, 7), viruses (8–10), and parasites (11). For example, classic experiments in which human volunteers ingested typhoid bacilli demonstrated the relationship between dose and the pathogenesis of typhoid fever (12, 13). However, such studies cannot ethically be done to understand the effect of inhaled dose and TB pathogenesis in humans. Our understanding of the effects of inhaled dose on the pathogenesis of TB is based largely on experimental work.

### Brief review of the history of the science of transmission of *M. tuberculosis*

Robert Koch conclusively demonstrated that *M. tuberculosis* is the etiologic agent of TB in 1882, but it was not until 1959 that Richard Riley and William Firth Wells and colleagues first demonstrated airborne transmission of *M. tuberculosis* to guinea pigs exposed to the air from humans with pulmonary TB in an experimental TB ward. Since then, TB has been considered the prototype disease of airborne transmission with a general acceptance that the other modes of transmission, i.e., large droplet and contact, do not occur. However, this is not absolute as there are reports indicating that the other modes of transmission rarely occur. Children can develop cutaneous TB in the form of chancres or warty TB from direct inoculation of bacilli onto damaged skin (14). A rare case of contact transmission occurred when a physician performed mouth-to-mouth resuscitation on a TB patient and acquired primary cutaneous TB in the nasolabial area (15). Also, direct injection of *M. tuberculosis* among pathologists and other laboratory workers can produce “prosectors warts,” i.e., granulomas at the site of accidental injection, first described by Laennec regarding a lesion on his own finger (16). Under our second hypothesis section later in this paper, we review the evidence for upper airway deposition of *M. tuberculosis* by large droplet aerosols.

**Figure 2:**
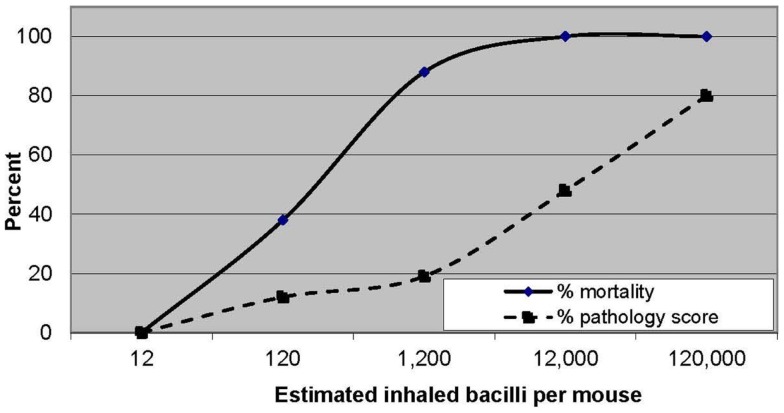
**Increasing mortality and pathological changes in mice with increasing inhaled doses of *Mycobacterium bovis***. Data from Ref. (18).

Koch was also the first to demonstrate a dose–response effect of infection on pathogenesis (17). His observation of the association of higher mortality with increasing inoculum size was later confirmed by Glover (18) and Youmans (19). Glover’s semi-quantitative estimate of inhaled dose was also associated with lung pathology findings, ranging from small granulomatous lesions in the mice exposed to the lowest doses of *M. bovis* to gross suppurative consolidation in those exposed to the highest doses of *M. bovis* (Figure 2).

Experimental aerogenic infections of the mouse, guinea pig, and rabbit models (20, 21) have demonstrated similarities in the kinetics of immunopathogenesis after infection. After infection in a given host that is not highly susceptible, growth occurs in the lung at a logarithmic rate for the first 2–3 weeks, and then plateaus at approximately 4 weeks when the infection is controlled by acquired immunity. In an immunologically naïve animal, more extensive replication of the bacteria is needed to induce the immune response, so the time course may be longer (approximately 3–4 weeks) than in the animal with pre-existing immunity (21). The genetic susceptibility of the animal determines the immune response to a given bacillary strain. For example, in Lurie’s studies of rabbits of varying susceptibility to infection, one resistant strain produced one grossly visible pulmonary tubercle per 600 viable H37Rv bacilli inhaled, whereas a susceptible strain developed one tubercle per 10^7^ bacilli (20). However, for a given strain of host rabbit and a given strain of tubercle bacillus, intensity of airborne dose was associated with an increased number or rabbits infected and with shorter survival time. Similarly, natural resistance to TB in the rabbit model can be overcome by larger inhaled doses of tubercle bacilli (22), suggesting a strong dose-response relationship between inoculum size and risk of disease.

More recent studies using the same strain of mouse (C57BL/6) exposed to aerosols of three different strains of *M. tuberculosis* demonstrated that there was more rapid progression of disease associated with increasing bacillary concentrations in the aerosol within each strain, although a similar rapid progression of disease was observed when comparing more to less virulent strains. In other words, high inhaled doses of less virulent strains may produce similar lung pathology as lower doses of less virulent strains (23). In addition to the course of TB disease, the inhaled inoculum size has recently been shown to influence the therapeutic response to antituberculous medications (24).

These animal models all reflect acute infection, or what in humans is termed “primary TB disease” or “progressive primary disease.” This occurs most commonly in children <5 years of age (25) or in HIV infected persons (26). However, in one large study of 20,687 household contacts (HHCs) age 5 years or older), 598 (4.9%) had primary TB (25).

### Models of LTBI

LTBI has been difficult to study due to the lack of good animal models until recently. The “Cornell Model” of LTBI involved the establishment of active TB disease that was then partially treated with antituberculous agents (27). The mice were then immunosuppressed to reactive TB disease in an effort to model the reactivation of TB in humans. Although some humans do reactivate LTBI due to immunosuppression, the vast majority of humans reactivate spontaneously without the medical intervention of immunosuppressive drugs. Why the majority of humans have LTBI but never reactivate has never been explained.

#### Non-human primate models

Although the experimental mouse model has provided valuable contributions to our understanding of the immunology of TB, it does not sufficiently model key aspects of human TB disease, notably necrotic lesions or cavitation or LTBI. Also, whereas the guinea pig and rabbit models have pathology more similar to humans, there are few immunologic reagents for these models. Some of the most exciting data from animal models in recent years has been generated using non-human primates (NHP) because of the similarity of TB disease to human disease and because of cross-reactivity of immunologic and molecular reagents with humans.

Early experiments used intratracheal administration of *M. tuberculosis* to Philippine cynomolgus monkeys (*Macaca fascicularis*) (28). Delivery of very high doses of 10^4^ or 10^5^ colony-forming units (CFU) of *M. tuberculosis* resulted in acute and rapidly progressive and fatal multilobar pneumonia. However, lower doses (<10^3^ CFU) produced a more chronic and slowly progressive form of pulmonary TB that was similar to human disease sometimes with dissemination to other organs. Even lower doses (10^1^ or 10^2^ CFU) resulted in some monkeys containing the infection in a subclinical state.

Another group of investigators has used bronchoscopic administration of *M. tuberculosis* suspensions to infect 17 cynomolgus macaques with a low dose of approximately 25 CFU (29). They observed a range of pathology, with 7 classified as LTBI and eight remained alive and well, without evidence of disease after 15–21 months of observation. The other animals exhibited a range of pathology similar to primary human TB, and one animal may have reactivated with a small cavitary lesion.

Natural human infection with *M. tuberculosis* most often occurs after inhalation of infectious aerosols and yet another group of investigators have demonstrated that macaques infected by the head-only aerosol route develop disease that appears to be very similar to human TB. High doses of aerosols of *M. tuberculosis* produce a fulminant fatal pneumonic TB in rhesus macaques (30, 31). Lower doses of about 500 CFU of CDC 1551 strain produced asymptomatic, latent infections in 12 animals except for one with signs of active TB over 9 weeks of observation (32). All 11 appearing to have LTBI had no evidence of *M. tuberculosis* on bronchoalveolar lavage (BAL) at 9 weeks. Six animals were infected intravenously with simian immunodeficiency virus (SIV), and they all developed culture-positive BALs, and some had evidence of dissemination with positive blood cultures. The resurgence of the NHP model of TB is in part due to the availability of immunologic reagents for the study of AIDS, and reactivation of TB infection produced by either bronchoscopic instillation or by aerosol exposures, and has been demonstrated due to co-infection of macaques with SIV (32, 33). The NHP model has also demonstrated reactivation of LTBI with inhibition of tumor necrosis factor (TNF) alpha similar to that in humans (34).

In summary, the NHP model of TB has demonstrated the importance of inhaled (or instilled) dose in the establishment of LTBI versus primary (progressive) TB disease. What is not known is whether dose is a predictor of which patients with LTBI will reactivate in the future. We propose the concept that exposure of humans to large concentrations of infectious aerosol results in both (1) an increased risk of inhalation of live bacilli that can establish a focus of infection and (2) increased opportunities for multiple episodes of inhalation of one or more bacilli that may establish multiple foci of infection. Given recent evidence about the dynamic nature of the establishment of infection in a NHP model (35), this would increase the chances that one of those foci would result in a progressive infection acutely, as well as reactivation of a focus of infection at a later time point.

## Epidemiological Evidence Supporting a Dose–Response Relationship

Epidemiologic studies have also suggested a dose–response relationship in TB immunopathogenesis. Epidemiologic studies of TB have been hampered by the inability to measure inhaled dose, or at least a measure of source strength from the index case with TB. This resulted in the use of the sputum smear as an indirect measure of bacillary load in the infectious aerosols generated by TB patients.

### Household contact studies

Our understanding of transmission of *M. tuberculosis* to humans has been largely determined by epidemiological studies of HHCs of patients with pulmonary TB and by reports of nosocomial outbreaks. Close contacts of persons with sputum-smear-positive TB have the highest prevalence of LTBI compared to other populations, and they are second only to HIV infection for the highest risk of progressing from LTBI to active disease (36, 37). Increased transmission has been consistently associated with sputum smear-positive cases compared to that of sputum smear-negative cases. This has been most well-documented by comparing TSTs in HHCs who are exposed to patients with sputa that is smear-positive for AFB versus those with sputa that is smear-negative for AFB but culture-positive for *M. tuberculosis*. For example, in a classic study done in Canada, there was a gradient of higher rates of infection and secondary TB disease cases among HHCs exposed to sputum smear-positive cases, HHCs compared to smear-negative cases, and community controls (38). Among close contacts who developed active TB, 92% were exposed to a smear-positive case and only 8% to a smear-negative case. In another large study done in Chennai, India, the 15-year risk of progression to disease was highest among HHCs of sputum smear-positive/culture-positive cases (adjusted RR = 3.4; 95% CI 3.0–3.9) compared to contacts of sputum smear-negative/culture-positive cases (aRR 1.7; 1.4–2.0) and community controls without household exposure to TB (39). In a more recent study conducted in Galicia, Spain during 1996–2011 (40, 41), there were 3822 cases of smear-negative TB, most of them identified in contact tracing. The frequency of LTBI in HHC of these patients was similar to contacts of smear-positive cases (30.1 versus 33.4%). By contrast, the frequency of TB disease in HHC of smear-negative cases was <20% that of smear-positive cases.

The observed effect of AFB smear-positivity is most likely a simple quantitative difference in the concentration of bacilli in the respiratory secretions of the source cases, as approximately 5,000– 10,000 bacilli per milliliter must be present for detection by direct microscopy with Ziehl–Neelsen staining (42).

The percentage of TST-positive contacts of sputum smear-positive cases has varied from 1.1 to 73%, and that of contacts of smear-negative cases has ranged from 0 to 46%, with community rates from <1 to 41%. In a community-based study in England between 1948 and 52, positive TSTs were found in 65.2% of children exposed to a smear-positive case and 26.8% exposed to a smear-negative case (43). A strength of this study was that cases of active TB disease were also tracked. Positive TSTs were found in 14.4% of children exposed to a smear-positive case and in 3% of children exposed to a smear-negative case. However, interpretation of these data must include the background or community prevalence of a positive TST, and these data may vary widely (44). Although the TST has been a useful tool, it has many limitations, not the least of which is the inability to detect when infection occurred (44, 45).

Furthermore, epidemiological data suggest that there is marked variability of transmission from sputum-based studies (46). For example, in a Dutch study of contacts to smear-positive index TB cases in five clinics in Rotterdam from 1967 to 69, only 22 of 80 (27.5%) index cases were found to have infected their contacts. However, when the distribution was stratified by age, 57% of index cases under 40 years of age infected their contacts but only 9% of the index cases over 60 did so (47). Our preliminary data from Florida also suggest that <30% of sputum smear-positive index cases transmit to HHCs (see below), consistent with these data. Moreover, approximately 15% of transmission in three different populations has been associated with sputum smear-negative disease (46).

### Intensity of exposure and TB outcomes

**Figure 3:**
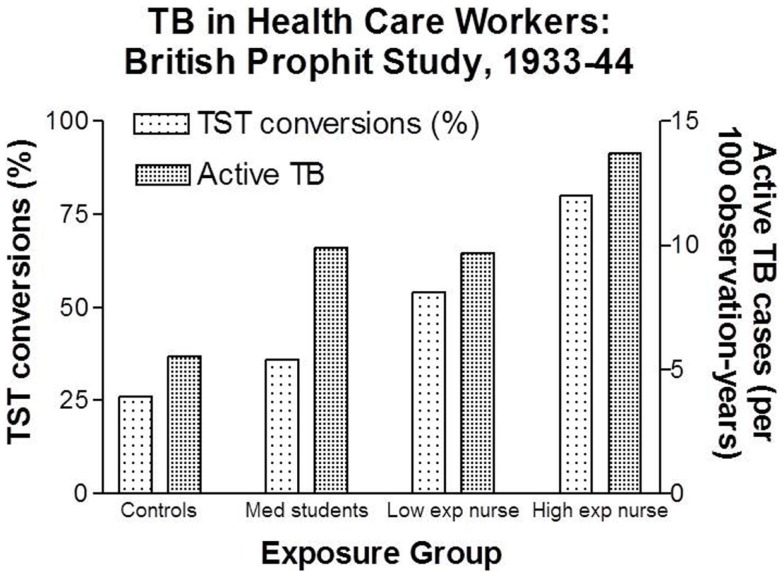
**Increasing rates of latent TB infection and active TB among health care workers with increasing degrees of estimated exposure to airborne *M. tuberculosis***. Data from Ref. (48).

Intensity of exposure can be considered a surrogate measure of inhaled dose due to the likelihood of larger and more frequent exposures to infectious aerosols. Probably the best data regarding the effect of intensity of exposure to *M. tuberculosis* on TB infection and disease were the British Prophit Study conducted between 1933 and 1944, a time when TB was highly prevalent in the UK (48). As the data in Figure 3 demonstrate, 80% of the nurses in the highest exposure group demonstrated conversions of their TSTs compared to 26% in community controls. The nurse cohorts were more similar to each other and, therefore, more comparable than the community controls and medical student. Compared to nurses working in low exposure settings, those working with TB patients in high exposure settings had a 48% increased risk of TST conversion and a 41% increased risk of active TB.

### Proximity of exposure and TB outcomes

Proximity can also be considered a surrogate for dose, as concentrations of airborne hazards are higher the closer a person is to an infectious source. The association of proximity of exposure and TB infection has been demonstrated in various settings. HHCs sharing the same bed are at higher risk of infection than those living in another room in the same residence (49–51). Patients exposed to the infectious aerosols generated by a forceful irrigation of a tuberculous abscess were found to be infected along a clear spatial gradient, with more patients infected with TB the closer their rooms were along the hospital hallway (52). Similarly, airline passengers on a transoceanic flight were more likely to have evidence of TB infection the closer they were sitting to an infectious TB patient (53). In a classic outbreak of TB aboard a U.S. Navy ship, proximity and duration of contact were associated with both TB infection and disease (54, 55). In an unusual exposure to aerosols generated by water jet irrigation of a tuberculous abscess, there was a clear gradient of infection associated with proximity (52). A limitation of the epidemiological data on proximity of exposure is that it is not possible to determine if there were simply more frequent opportunities to become infected with similar inhaled doses or if there were greater chances of being exposed to larger concentrations of *M. tuberculosis* aerosols. Without a better surrogate measure for inhaled dose, such questions cannot be answered.

### Transmission of *M. tuberculosis* and cough

In addition to the bacillary load in the sputum, another known risk factor for transmission is the frequency of cough. In Oslo from 1940 to 53, the presence of cough in TB patients with smear-positive sputa increased the risk of infections in exposed children <5 years old (19.7 versus 5.5% in those without cough) (56). IN another study, Loudon recorded 8-h overnight cough frequency in newly admitted TB patients. Positive TSTs were found in 44% of contacts of patients who had more than 48 coughs per 8 h period compared to 28% of contacts of patients who had <12 coughs per night (57). In a more recent study in Brazil, we found that cough assessed by a visual analog scale or the Leicester Cough Questionnaire was associated with more transmission (58). Epidemiological studies have found that more secondary cases of TB are associated with men than with women, and that they decrease with advancing age (59). One explanation for this finding is that young patients tend to have more close contacts and more contacts in general than do older patients. However, we suggest that another explanation may be that younger persons and men may have higher forced expiratory volumes and thus be able to generate more cough aerosols, as we observed among patients with cystic fibrosis (CF) (60). Our preliminary data discussed below support the importance of cough in generating infectious aerosols of *M. tuberculosis*.

## Cough Aerosol Cultures as a Surrogate for Inhaled Dose

While the inhaled dose can be manipulated in experimental animal models or *in vitro* systems, it is not possible to measure in human infections. It is obviously unethical to deliberately expose humans to varying concentrations of infectious aerosols of *M. tuberculosis*, as has been done with less virulent diseases. So any attempts to estimate inhaled dose must be observational and retrospective in nature, as human patients present either after they have become ill with active TB or after they have become exposed to someone with active TB. Thus, we must use markers that are surrogates for the inhaled dose.

### Measurement of *M. tuberculosis* in cough-generated aerosols

Although aerosol transmission of TB was convincingly established over 50 years ago, microscopy of sputum smears for AFB continues to be used as the best laboratory test of infectiousness due to the lack of better options. As discussed, whereas the sputum AFB smear has been a useful surrogate marker of inhaled dose for over 100 years, it clearly has its limitations and should only be considered a risk factor for infectiousness (61, 62). There is a need and capacity for a better surrogate marker of infectiousness.

During the resurgence of TB in the U.S. in the late 1980s and early 1990s, significant morbidity and mortality among health care workers (HCWs) was recognized. The use of personal respiratory protection (PRP, e.g., N95 respirators) was especially controversial, and the efficacy of PRP depends upon the particle size distribution of the inhalation hazard, in this case that of infectious aerosols containing *M. tuberculosis*. In addition, the size distribution of infectious aerosols determines how long they will remain airborne and where in the respiratory tract they are most likely to deposit. Although Wells had estimated the size range of infectious droplet nuclei to be 1–5 microns based on experimental nebulization in the laboratory and theoretical principles, the size of infectious aerosols of *M. tuberculosis* had never been directly measured. To address this need, we developed a “cough aerosol sampling system (CASS)” and initially aimed to study cough aerosols from patients with TB to determine the magnitude and particle size distribution of infectious aerosols of *M. tuberculosis* to improve the scientific basis for interventions to protect HCWs.

In our initial feasibility study using CASS in 16 AFB-positive patients at a referral hospital in the U.S., we collected culturable aerosols from only 4 (25%) of them (63). Aside from the small sample size and preliminary nature of that work, one criticism was that the sampling was done too close in proximity to the patient, and thus may not represent what infects human patients (64). However, this is probably not the case, as airborne droplets desiccate to the size of infectious droplet nuclei in <1 s (65, 66). Others more informally suggested that the method was too highly technical to be done in high incidence settings such as sub-Saharan Africa. We subsequently used the CASS to collect cough aerosols from sputum AFB-smear-positive TB patients in Kampala, Uganda (67). We cultured *M. tuberculosis* from the cough aerosols of 28 of 101 (27.7%; 95% confidence interval [CI], 19.9–37.1%) patients with culture-confirmed TB, with a median 16 CFU (range, 1–701) in 10 min of coughing. Nearly all (96.4%) cultivable particles were 0.65–4.7 μ in size, in agreement with Wells’ theoretical predictions decades earlier. Positive aerosol cultures were associated with higher physical performance (Karnofsky) scores (*P* < 0.016), higher sputum AFB smear microscopy grades (*P* < 0.007), lower days to positive in liquid culture (*P* < 0.004), stronger cough (*P* < 0.016), and fewer days on TB treatment prior to aerosol collection (*P* < 0.047). In multivariable analyses, cough aerosol cultures were associated with a salivary/mucosalivary (compared with purulent/mucopurulent) appearance of sputum (odds ratio, 4.42; 95% CI, 1.23–21.43) and low days to positive in liquid culture (per 1 day decrease; odds ratio, 1.17; 95%CI, 1.07–1.33). This study demonstrated the feasibility of using this research method in a resource-limited setting and again suggested considerable variability in the ability to produce infectious aerosols among TB patients. These data suggest that although the bacillary load in sputum is one of the predictors of cough aerosol cultures, sputum bacillary load is not the only predictor. As noted by CDC investigators 30 years ago, the bacillary load in the sputum should be considered a risk factor for transmission, not the sole definition of infectiousness (62).

**Figure 4:**
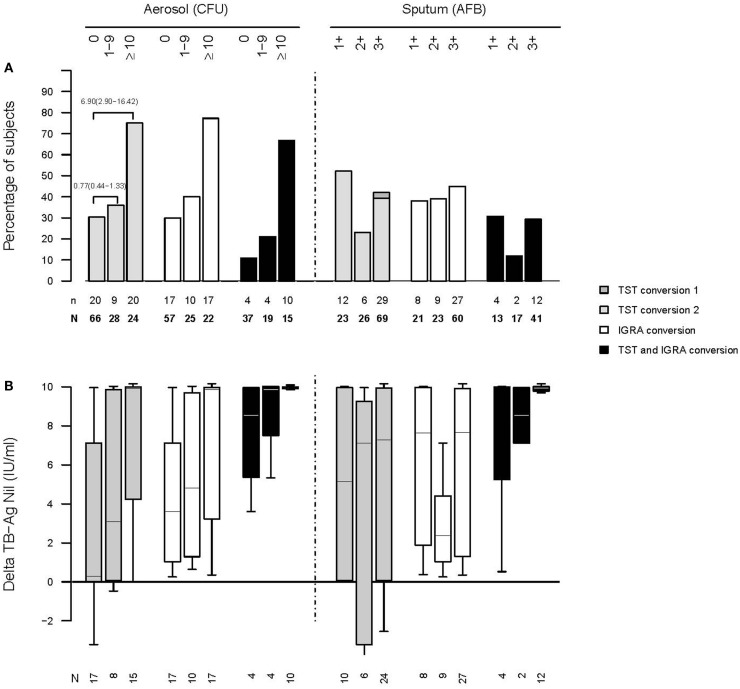
**Increasing rates of infection among household contacts of active cases of TB who produced larger cough aerosols (A)**. Increasing amounts of interferon gamma output in an interferon-gamma release assay among household contacts exposed to active cases of TB who produce larger cough aerosols **(B)** [from Ref. (68); please see that paper for details regarding numbers of subjects listed].

We followed up this study with another at the same site, this time linking cough aerosol cultures from the index TB cases to new infections among their HHCs, the primary outcome being TST conversion (68). We enrolled 96 sputum culture-positive index TB cases and their 442 contacts. Contacts of patients with TB who produced high aerosols (>10 CFU) were more likely to have a new infection compared with contacts from low-aerosol (1–9 CFU) and aerosol-negative cases (83%, 32%, and 30%, respectively; *P* < 0.009). A high-aerosol patient with TB was the only predictor of new *M. tuberculosis* infection in unadjusted (odds ratio, 6.90; 95% confidence interval, 6.90, 16.42) and adjusted analyses (odds ratio, 4.81; 95% confidence interval, 1.20–19.23). In other words, when cough aerosols were included in models, the traditional indicators of infectiousness such as AFB sputum smear, culture, or cavitation were no longer significant (Figure 4).

In addition to these data, the use of cough aerosols as the best indicator of infectiousness is supported by Hill’s guidelines to strengthen the inference that a statistical association is also causal (69). There is clearly more biological plausibility favoring cough aerosols over sputum, as we know that pulmonary TB is not transmitted by sputum but rather through aerosols, i.e., infectious particles in the air. The experimental animal model evidence reviewed above favors cough aerosols over sputum, although a head-to-head comparison of the AFB sputum smear and cough aerosol cultures has not been done. However, this can be tested relatively easily using the approach of natural infection of guinea pigs (64). Temporality is a difficult criterion to satisfy for any surrogate, including sputum, necessarily obtained after infection has occurred. The strength of the association now favors cough aerosols given the data presented above, although it would be ideal for those data to be replicated in other settings by other investigators. Thus, there has not yet been enough data generated to evaluate consistency. Finally, we have argued above that there is a gradient, or dose–response, so we suggest that cough aerosols are the best surrogate for inhaled dose.

Why should *M. tuberculosis* be one of the few pathogens which is transmitted exclusively by the airborne route? We suggest that one reason is that airborne transmission of other pathogens has not been well studied. There is a growing body of literature indicating that influenza is probably at least partially transmitted by the airborne route as well as by large aerosols and direct contact (70). *Pseudomonas aeruginosa* and other Gram-negative bacilli (GNB) have been thought to be transmitted only by large droplet transmission, but coughing CF patients readily produce viable aerosols of these opportunistic pathogens (60, 71). However, transmission of disease via the fine aerosols of these GNB has not yet been demonstrated. It is most likely that many pathogens can be aerosolized from the human respiratory tract by coughing or sneezing. Whether or not transmission occurs is dependent on the concentration at the time of emission from the index case and subsequent dilution before inhalation by the exposed host as well as survival of the immediate desiccation and temperature change, exposure to ultraviolet light, and other environmental stresses during transport (72).

**Figure 5:**
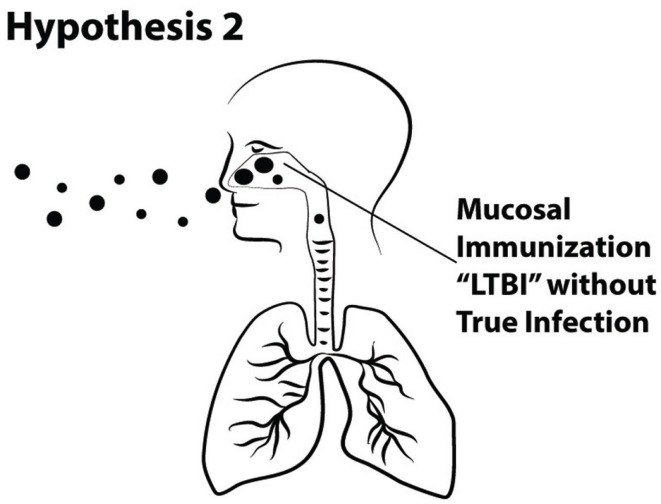
**Schematic of Hypothesis 2 demonstrating immunization via the mucosa of the upper airway without the establishment of true TB infection in the alveolar region of the lung**.

Hypothesis 2. Large droplet aerosols (>5 μ) of *M. tuberculosis* that deposit in the upper airway can induce immune responses without establishment of infection (Figure 5).

Although the discovery that TB is transmitted by the airborne route was brilliant, we respectfully suggest that the assumption that it is transmitted only by the airborne route is illogical. In this section, we argue that although TB disease is clearly preferentially transmitted by the airborne route, this is not exclusive or obligate. Furthermore, sensitization or immunization may occur due to deposition and presentation of tuberculous antigens in the upper airway, without causing persistent infection or disease.

### A short history of the science of TB transmission mechanisms

Cornet (a student of Robert Koch) inoculated guinea pigs with bacilli from the dust from the rooms of TB patients and was able to produce infection in 40 of 118 samples in one experiment and in 15 of 21 samples from medical wards, demonstrating that tubercle bacilli can remain infectious for long periods of time. The fact that *M. tuberculosis* can be found in dust attests to the fact that large droplets from patients settle to the ground. Flugge doubted the role of dust and appears to be the first to consider cough aerosols as a primary mechanism of transmission of TB. He sprayed dried versus moist sputum of TB patients to create aerosol exposures of guinea pigs and it was only those exposed to the moist sprays that became infected, and he had patients cough into the air, exposing guinea pigs. On finding that droplets on settle plates between the patients and the guinea pigs grew *M. tuberculosis*, he concluded (we now know incorrectly) that these large droplets caused TB (73). However, these early experiments indicated that large droplet aerosols from TB patients contained viable *M. tuberculosis*.

Another early line of evidence indicating large droplet transmission was the observation that *M. tuberculosis* could be cultivated from the nasal cavities of 9 out of 29 healthy hospital workers with no evidence of TB disease (74). This is similar to another potentially lethal respiratory infection, inhalation anthrax, that was historically an occupational disease associated with exposures to wools and other materials contaminated with *Bacillus anthracis*. It was common to find anthrax spores in anterior nasal swabs and pharyngeal washings in exposed workers who had no clinical symptoms or signs of disease (75).

### High rates of reactivation of LTBI

An accepted paradigm in the science of TB is that only 10% of persons infected with *M. tuberculosis* will develop TB disease, half of those developing disease within the first few years of exposure and the others at a later point in their lives. However, we are unable to find good explanations for why 90% of infected humans never develop disease. More recently, Horsburgh and colleagues evaluated data from the United States, and they found a low overall number of cases developing within the first 5 years after a positive TST (76). Overall, there were only 27 TB cases among 7742 persons tested, or 0.35% over 5 years. However, among the 106 HIV infected persons in that study, there were 12 cases, or 11.3% over 5 years. This represents the highest rate of reactivation studies.

Although HIV infection has been associated with high rates of reactivation of TB, the use of TNF-alpha inhibitor drugs has also been associated with a large amount of disease reactivation. Early reports were case series that did not include data on baseline TSTs or IGRAs (77). In a report from France on 69 cases of TB associated with the use of TNF-alpha inhibitors, there were 15 TB cases among the 38 persons (39.5%) with TSTs of 5 mm or more.

So if we accept TNF inhibition as the strongest inducer of reactivation TB, even more so than HIV, why do we not see more than 40% develop active TB disease, especially when nearly all NHPs who are experimentally infected and then treated with TNF-alpha inhibition reactivate (34)? We propose one explanation is that some people are exposed to large droplet aerosols of *M. tuberculosis* that deposit in their upper airway and induce an immune response but do not cause infection. The traditional test defining LTBI has been the TST, but it reflects delayed hypersensitivity to the antigens of *M. tuberculosis* and is only a surrogate for cell-mediated immunity (CMI). The more recently developed IGRAs are probably a better reflection of CMI. However, in the discussion to follow, we suggest that both delayed hypersensitivity and CMI may be stimulated without a true infection occurring.

## Cough Aerosol Particle Size Distribution in Health and Disease

Aerosols are suspensions of particles in air (65, 78).The most important determinant of particle deposition within the respiratory tract is the mass median aerodynamic diameter (MMAD), or the diameter of a particle if it behaved as a sphere (65, 78). For simplicity, we will simply use the term “diameter.” Most aerosols in nature contain a wide range of sizes, or they are “polydisperse.” “Monodisperse aerosols” of a single diameter can generally only be produced in the laboratory.

### Cough aerosols from healthy humans

**Figure 6:**
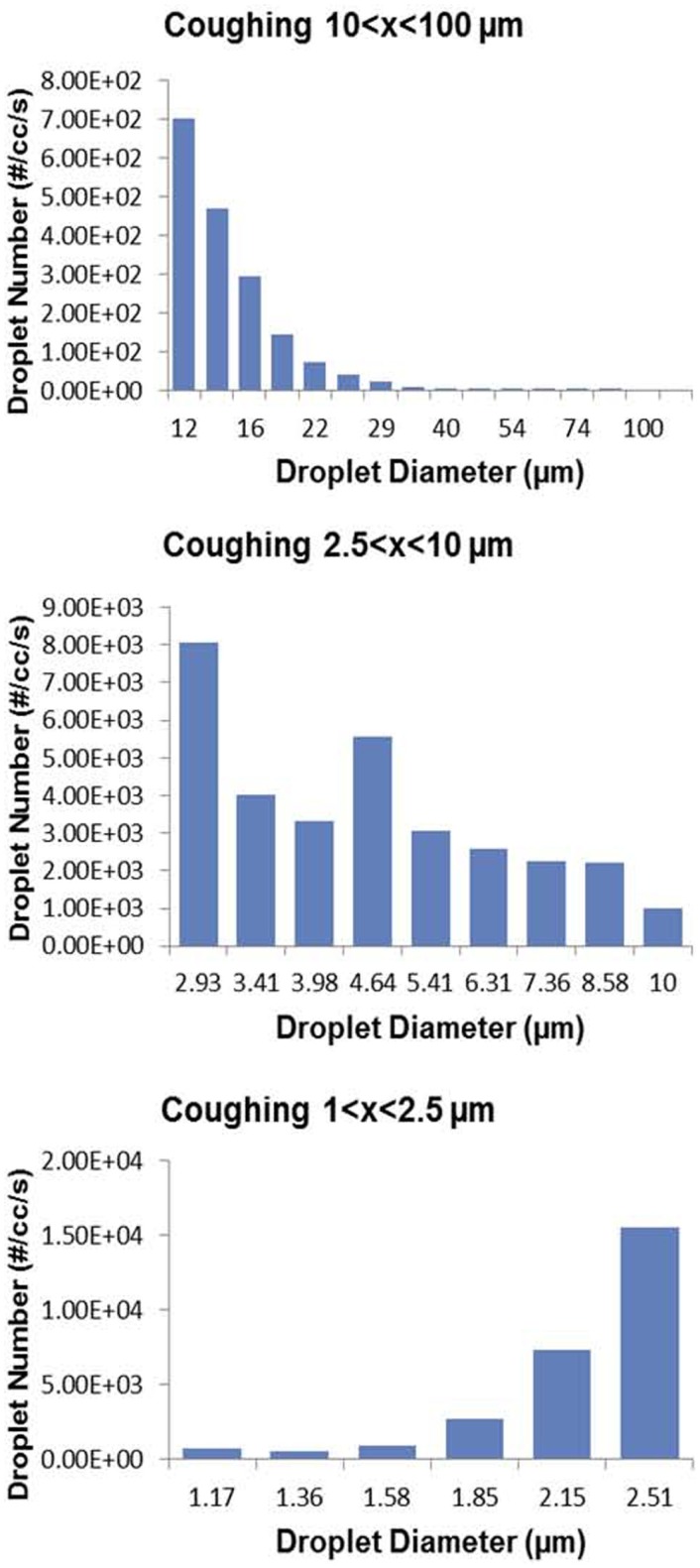
**Wide range of particle size distributions of aerosols produced by voluntary coughing by healthy subjects**. From Ref. (80).

Most attempts to study the aerosols generated from the human respiratory tract have used healthy volunteers. In early studies, investigators applied dyes to the oral cavity and then measured the large droplets from the “mouth spray” on oiled slides held a few inches from the mouth; the size of smaller droplets were estimated by calculations from the larger ones. There was a wide range of particles from 1 to 2000 μ (79) In a more recent study, aerosols from five healthy human volunteers that were mouth breathing, nose breathing, coughing, and talking were measured using an optical particle counter and an analytical transmission electron microscope (80). Coughing produced the largest concentrations of droplets, but there was considerable variability. Most recently, Zayas and colleagues used a laser diffraction system to study 45 healthy subjects and found that voluntary coughs generated particles from 0.1 to 900 μ in size (81) (Figure 6).

### Cough aerosols during pulmonary infections

**Figure 7:**
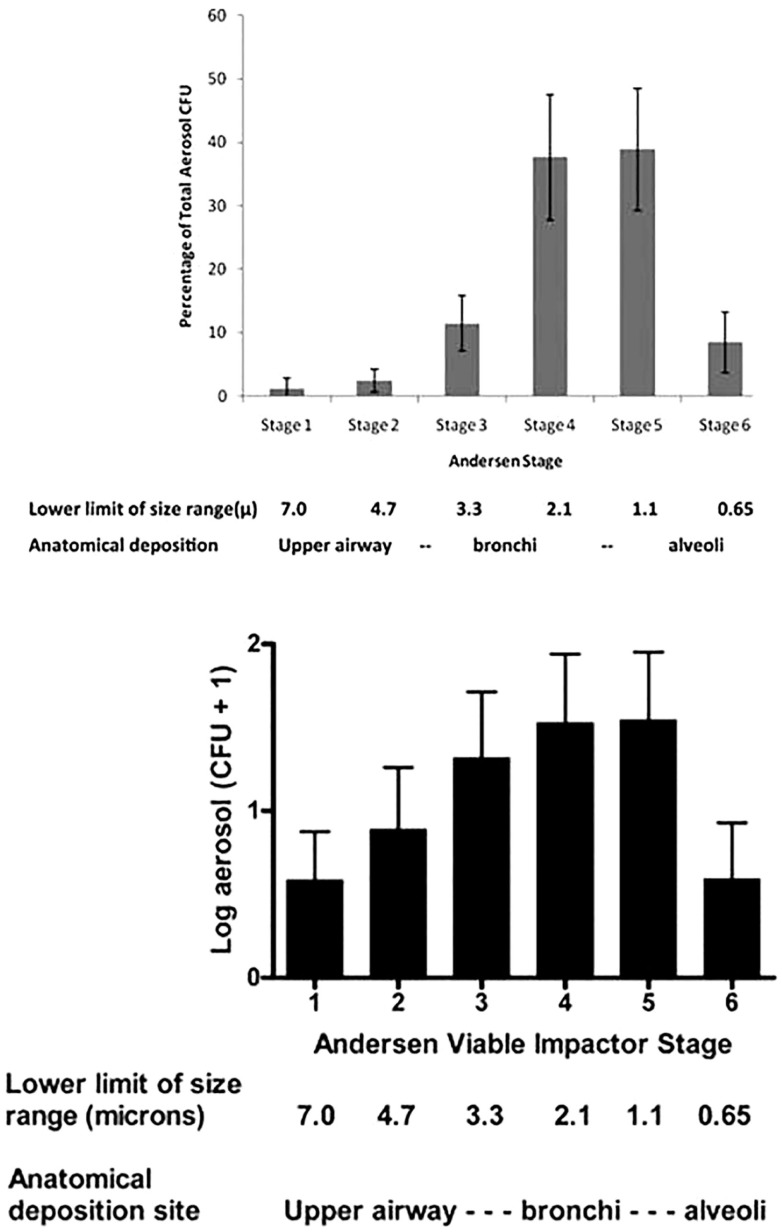
**Particle size distributions of cough aerosols from patients with active pulmonary TB [top panel; from Ref. (67)] and from cystic fibrosis patients with chronic *P. aeruginosa* infections [bottom panel; from Ref. (60)]**.

While the above data from healthy humans provide valuable insights into the particle sizes that can be emitted from the human respiratory tract, healthy humans do not cough and do not produce sputum. These data did not inform the size of the particles in which pathogens were emitted and transported. The classic and elegant early studies by Flugge, Riley, Wells, Lurie, and others determined that aerosols were generated from TB patients, but to our knowledge the particle size distribution had never been measured directly. The CASS apparatus discussed above is a method of quantifying and sizing cough aerosols (63), and we found that nearly all (96.4%) particles with culturable *M. tuberculosis* in cough aerosols from patients with active pulmonary TB were <4.7 μ (67). In a study of patients with CF who were infected with *P. aeruginosa*, we found that 72% of the cough aerosol particles containing culturable *P. aeruginosa* were 3.3 μ or less (60), with a distribution similar to that of TB patients (Figure 7). However, we also found culturable bacilli in the connector tubing between the patient’s mouth and the sampling chamber (60), suggesting carriage of bacilli in large droplets. In a follow-up study of 19 CF patients at the same center, they all produced culturable aerosols that were again markedly variable among the subjects and mostly in particles <3.3 μ. In addition, culturable *P. aeruginosa* was isolated at 4 meters and persisted in the air for 45 min (71).

Cough aerosols from patients with influenza have been measured more recently. In one study using Andersen cascade impactors as used for our TB and CF studies about, up to 89% of particles one foot away from patients were <4.7 μ (11% were larger) (82). In another study using different sampling methods of cough aerosols, influenza was found in 42% of particles <1 micron, in 23% of particles of 1–4 μ, and in 35% of particles >4 μ (83). In all of these studies, the sampling methods for viable pathogens are technically limited to the smaller size ranges, so our knowledge of the concentration of viable pathogens in large droplets is limited.

In summary, in both health and disease, humans generate cough aerosols in a broad range of particle sizes. Most pathogens are found in particles <4–5 μ, so predictably deposit in the lower respiratory tracts. However, pathogens, including *M. tuberculosis*, are also present in larger particles that probably deposit in the upper airways.

## Primary TB of the Upper Airway

Given the above discussion about the size distribution of cough aerosols from patients with pulmonary TB, it should not be surprising that *M. tuberculosis* can deposit in the upper airways. Most nasal TB occurs in conjunction with pulmonary TB. Primary nasal TB, by definition without co-existing pulmonary infection, is rare, but there are multiple case reports (84–88). Laryngeal TB almost always occurs in tandem with pulmonary TB, but primary TB of the larynx occurs, probably more rarely than primary nasal TB (89–93).

Primary oral TB is also rare, although *M. tuberculosis* may be cultured from the dental plaque and saliva of patients with pulmonary TB (94). In a study of TB patients with periodontal disease, 92% had *M. tuberculosis* isolated in the saliva and 68% in the dental plaque. In this endemic setting in India, 12% of periodontitis patients not diagnosed with TB also had *M. tuberculosis* isolated from the saliva (95). In an unusual outbreak, a dentist with pulmonary TB had presumably coughed into the mouths of the cases or contaminated them with *M. tuberculosis* on his fingers, resulting in 13 cases of primary intraoral TB and involvement of regional lymph nodes, 1 case of both intraoral and pulmonary TB, and 1 case of erythema nodosum and pleural effusion (96). In several of these case reports, clinicians have suspected that nose-picking and smoking/alcohol use are risk factors for primary nasal and laryngeal TB, respectively. In the case of the outbreak of primary intraoral TB, dental manipulation or tooth extractions could obviously damage the oral mucosa. The suspected need for damaged mucosa is supported by a study using organ cultures of human tissue in which *M. tuberculosis* only adhered to extracellular matrix in areas of mucosal damage, but not to ciliated mucosa, intact extruded cells, basement membrane, or collagen fibers (97). We suspect that the rarity of primary TB of the upper airway is explained by the requirement of both damaged mucosa as well as deposition of large aerosol particles from a TB patient.

### Cervical lymphadenitis

The cervical lymph nodes are the most common site of TB lymphadenitis. Cervical lymphadenitis due to *M. tuberculosis* is thought by some authors to be a “local manifestation of a systemic disease,” presumably via lymphatic drainage from pulmonary lymph nodes (98). However, it is also clear that cervical nodes may become involved due to tuberculous infection in the head or neck (99). In a study of 100 cases of histologically confirmed TB cervical lymphadenitis, the most common site of disease was in the posterior triangle (51%), followed by the upper deep cervical (48%) and submandibular nodes (100). The supraclavicular and lower deep cervical nodes were involved in only 3 and 9%, respectively. These data suggest that an unappreciated source of infection of upper cervical lymph nodes is the upper airway. Cervical lymphadenitis from non-tuberculous mycobacteria, most common in children, is assumed to be due to a portal of infection in the oropharynx or conjunctiva (101). We propose that the same is likely to be true for *M. tuberculosis* if we accept that it is found in large aerosol droplets.

## Mucosal Immunization via the Upper Airway

Patients who suffer from “hay fever” (allergic rhinitis) are all too familiar with the immunological hypersensitivity response after airborne particles of pollen (that are usually around 20 μ or larger, similar to large droplet aerosols) deposit in the nose. The mucosa of the upper airway is a rich connection between the environment and the immune system. There is a rapidly growing literature on the use of intranasal vaccination, also known as mucosal immunization. Intranasal administration of vaccines provides protection against a variety of respiratory pathogens (102). Intranasal vaccination with *M. bovis* BCG can elicit not only protection against aerosol challenge with *M. tuberculosis* H37Rv, but it can also elicit granuloma formation in the deep lung (103). Even heat-killed BCG delivered intranasally with an adjuvant can induce systemic immune responses (104). Intranasal administration of BCG to mice appears to offer short-term protection in the lung but persistent immune responses in the spleen for at least 10 months (105). We speculate that this may help explain the poor ability of BCG vaccination to protect humans from pulmonary TB but the documented protection against disseminated disease.

## Summary

An inoculum effect is common in most infectious diseases, and we have reviewed data demonstrating the greater likelihood of more progressive TB disease with greater magnitudes of inhaled infectious aerosol or with surrogate measures for the same. We propose that cough aerosols of *M. tuberculosis* are the best surrogate measure to test our first hypothesis that inhaled dose predicts immunopathology in human TB. Our second hypothesis, that large droplet aerosols of *M. tuberculosis* can induce immune responses without establishing infection can be tested in both experimental animal models and perhaps in the near future in humans with attenuated live mycobacteria or with mycobacterial antigens. Such research would have the potential to discover better biomarkers of true persisting infection, and hopefully, to better identify those at risk for progression to active TB.

## Author Contributions

KF and EJ-L both conceived of this work and contributed to the review of the literature; both contributed to drafts, revisions, and the final manuscript; both are accountable for all work and agree to the final manuscript.

## Conflict of Interest Statement

The authors declare that the research was conducted in the absence of any commercial or financial relationships that could be construed as a potential conflict of interest.
