# Corrigendum: Quantity and Quality of Inhaled Dose Predicts Immunopathology in Tuberculosis

**DOI:** 10.3389/fimmu.2015.00511

**Published:** 2015-10-01

**Authors:** Kevin P. Fennelly, Edward C. Jones-López

**Affiliations:** ^1^Department of Medicine, Emerging Pathogens Institute, University of Florida, Gainesville, FL, USA; ^2^Section of Infectious Diseases, Boston Medical Center, Boston University School of Medicine, Boston, MA, USA

**Keywords:** tuberculosis, inhaled dose, pathology, inoculum, immune response

We apologize for the incorrect citations of certain references that occurred in the reference list of our original article.

We have corrected the references below.

8. Alberti A, Chemello L, Benvegnu L. Natural history of hepatitis C. *J Hepatol* (1999) **31** (Suppl 1):17–24.

9. Friedland GH, Klein RS. Transmission of the human immunodeficiency virus. *N Engl J Med* (1987) **317**:1125–35. doi:10.1056/NEJM198710293171806

10. Quinn TC, Brookmeyer R, Kline R, Shepherd M, Paranjape R, Mehendale S, et al. Feasibility of pooling sera for HIV-1 viral RNA to diagnose acute primary HIV-1 infection and estimate HIV incidence. *AIDS* (2000) **14**:2751–7.

11. Pereira BA, Alves CR. Immunological characteristics of experimental murine infection with *Leishmania* (*Leishmania*) *amazonensis*. *Vet Parasitol* (2008) **158**:239–55. doi:10.1016/j.vetpar.2008.09.015

12. Hornick RB, Greisman SE, Woodward TE, DuPont HL, Dawkins AT, Snyder MJ. Typhoid fever: pathogenesis and immunologic control. 2. *N Engl J Med* (1970) **283**:739–46. doi:10.1056/NEJM197010012831406

23. Rhoades ER, Frank AA, Orme IM. Progression of chronic pulmonary tuberculosis in mice aerogenically infected with virulent mycobacterium tuberculosis. *Tuber Lung Dis* (1997) **78**:57–66.

24. de Steenwinkel JE, ten Kate MT, de Knegt GJ, Verbrugh HA, van Belkum A, Hernandez-Pando R, et al. Course of murine tuberculosis and response to first-line therapy depends on route of infection and inoculum size. *Int J Tuberc Lung Dis* (2011) **15**:1478–84. doi:10.5588/ijtld.11.0012

39. Radhakrishna S, Frieden TR, Subramani R, Santha T, Narayanan PR. Additional risk of developing TB for household members with a TB case at home at intake: a 15-year study. *Int J Tuberc Lung Dis* (2007) **11**:282–8.

51. Rathi SK, Akhtar S, Rahbar MH, Azam SI. Prevalence and risk factors associated with tuberculin skin test positivity among household contacts of smear-positive pulmonary tuberculosis cases in Umerkot, Pakistan. *Int J Tuberc Lung Dis* (2002) **6**:851–7.

The original article has been updated.

## Conflict of Interest Statement

The authors declare that the research was conducted in the absence of any commercial or financial relationships that could be construed as a potential conflict of interest.

